# Hemokinin-1 as a Mediator of *Arthritis*-Related Pain via Direct Activation of Primary Sensory Neurons

**DOI:** 10.3389/fphar.2020.594479

**Published:** 2021-01-13

**Authors:** Éva Borbély, Ágnes Hunyady, Krisztina Pohóczky, Maja Payrits, Bálint Botz, Attila Mócsai, Alexandra Berger, Éva Szőke, Zsuzsanna Helyes

**Affiliations:** ^1^ János Szentágothai Research Centre and Centre for Neuroscience, University of Pécs, Pécs, Hungary; ^2^ Department of Pharmacology and Pharmacotherapy, Medical School, University of Pécs, Pécs, Hungary; ^3^ Department of Pharmacology, Faculty of Pharmacy, University of Pécs, Pécs, Hungary; ^4^ Department of Medical Imaging, Medical School, University of Pécs, Pécs, Hungary; ^5^ Department of Physiology, Semmelweis University, Budapest, Hungary; ^6^ Princess Margaret Cancer Centre, University Health Network, Toronto, ON, Canada; ^7^ PharmInVivo Ltd., Pécs, Hungary

**Keywords:** experimental arthritis, arthritic pain, primary sensory neuron, neuroinflammation, tachykinin, *in vivo* optical imaging

## Abstract

The tachykinin hemokinin-1 (HK-1) is involved in immune cell development and inflammation, but little is known about its function in pain. It acts through the NK1 tachykinin receptor, but several effects are mediated by a yet unidentified target. Therefore, we investigated the role and mechanism of action of HK-1 in arthritis models of distinct mechanisms with special emphasis on pain. Arthritis was induced by i.p. K/BxN serum (passive transfer of inflammatory cytokines, autoantibodies), intra-articular mast cell tryptase or Complete Freund’s Adjuvant (CFA, active immunization) in wild type, HK-1- and NK1-deficient mice. Mechanical- and heat hyperalgesia determined by dynamic plantar esthesiometry and increasing temperature hot plate, respectively, swelling measured by plethysmometry or micrometry were significantly reduced in HK-1-deleted, but not NK1-deficient mice in all models. K/BxN serum-induced histopathological changes (day 14) were also decreased, but early myeloperoxidase activity detected by luminescent *in vivo* imaging increased in HK-1-deleted mice similarly to the CFA model. However, vasodilation and plasma protein extravasation determined by laser Speckle and fluorescent imaging, respectively, were not altered by HK-1 deficiency in any models. HK-1 induced Ca^2+^-influx in primary sensory neurons, which was also seen in NK1-deficient cells and after pertussis toxin-pretreatment, but not in extracellular Ca^2+^-free medium. These are the first results showing that HK-1 mediates arthritic pain and cellular, but not vascular inflammatory mechanisms, independently of NK1 activation. HK-1 activates primary sensory neurons presumably via Ca^2+^ channel-linked receptor. Identifying its target opens new directions to understand joint pain leading to novel therapeutic opportunities.

## Introduction

Rheumatoid arthritis (RA) is the most common autoimmune disorder of the joints characterized by chronic inflammation and severe pain. Although the inflammation can be effectively controlled by nonsteroidal anti-inflammatory drugs (NSAIDs), steroids, disease-modifying antirheumatic drugs (DMARDs) and biologic agents (Sparks, 2019), pain is often resistant to these drugs (McWilliams and Walsh, 2019). Persistent pain has resulted in an increased use of opioids among RA patients (Day and Curtis, 2019), though opioids are not effective in all cases (Chancay et al., 2019) suggesting more complex pain pathomechanisms in RA and making pain management an unmet medical need.

The joints are densely innervated by capsaicin-sensitive peptidergic sensory nerves (Donaldson et al., 1995) expressing, among others, the transient receptor potential vanilloid 1 (TRPV1) and ankyrin 1 (TRPA1) ion channels activated by a broad range of inflammatory mediators (Pinho-Ribeiro et al., 2017). These nerves play an important role in complex neuro-vascular-immune interactions resulting in chronic pain (Sun and Dai, 2018). Recently, intensive investigations have been initiated to reveal sensitization processes at molecular (Sommer and Kress, 2004) and histological levels alike (Ebbinghaus et al., 2019) that convert inflammatory to neuropathic pain and contribute to persistent arthritic pain. Exploration of its pathophysiological processes is hindered by the fact that no single animal model can mimic every aspect of RA, conclusions drawn using different models might not necessarily apply to the human disease (Krock et al., 2018). Studying the role of endogenous molecules of the sensory-vascular-immune interactions is essential to identify key mediators and potential novel drug targets.

Tachykinins are a family of neuropeptides that have been shown to play important roles in immune mechanisms, inflammatory vascular changes and pain (Onaga, 2014). Their best-known member, substance *p* (SP) acts mainly through the tachykinin neurokinin-1 receptor (NK1R), but can also bind to the other two tachykinin receptors, NK2R and NK3R, with much lower affinities. NK1R plays a key role in the wind-up mechanism in the spinal dorsal horn (Herrero et al., 2000), which is a crucial element in central sensitization leading to chronic pain. SP through NK1R also sensitizes the peripheral terminals of nociceptors (Nakamura-Craig and Smith, 1989). NK1R antagonists were developed as analgesic drug candidates, but despite promising preclinical results, they did not prove to be effective in human pain conditions. Therefore, tachykinins fell outside the focus of pain research until the discovery of hemokinin-1 (HK-1) in 2000 (Zhang et al., 2000) beginning a new era in this field. HK-1, encoded by the preprotachykinin-4 gene (*Tac4*), was first isolated from bone marrow B-cells (Zhang et al., 2000), and plays a role in T-lymphopoesis as well (Zhang and Paige, 2003). HK-1 consists of 11 amino acids, it has a close structural resemblance to SP, with seven matching aminoacids. HK-1 has the highest affinity to the NK1R similarly to SP (Morteau et al., 2001), but it has been proven that HK-1 can exert effects through other, so far unidentified target(s) (Borbély and Helyes, 2017). Our research group was the first to describe the mediator role of HK-1 in the chronic adjuvant-induced mouse model of inflammation and related nociception (Borbély et al., 2013). Recently, we proved that HK-1 is involved not only in inflammatory, but also nerve ligation-induced neuropathic pain, which develops independently of inflammation with prominent central sensitization mechanisms (Hunyady et al., 2019). The present work focused on investigating the involvement of HK-1 in arthritis models of distinct mechanisms with special emphasis on pain, nociceptive sensory neurons and the molecular mechanism of action.

## Materials and Methods

### Animals and Ethics

Experiments were performed on inbred 12–18-week-old (25–30 g) male *Tac4* gene-deficient (*Tac4* ^*−/−*^) knockout (KO) and NK1 receptor-deleted (*Tacr1* ^*−/−*^) mice and their C57BL/6J wild type controls (WT) purchased from Charles-River Ltd. (Hungary). The original *Tacr1* ^*−/−*^ breeding pairs (De Felipe et al., 1998) were obtained from the University of Liverpool, United Kingdom, The *Tac4* ^*−/−*^ strain was donated by A. Berger (Berger et al., 2010). Both KO strains were generated on C57BL/6J background and backcrossed to homozygosity for over five generations. The mutated allele’s germline transmission and excision of the selection cassette were verified by PCR analysis. The experimenters were blinded from the genotype and treatments in all cases. Animals were bred and kept in the conventional Laboratory Animal House of the Department of Pharmacology and Pharmacotherapy, University of Pécs at 24–25°C, 12 h light/dark cycles on wood shaving bedding in a standard polycarbonate cage with two to six mice per cage and provided with standard rodent diet and water ad libitum.

All experiments were carried out according to the European Communities Council Directive of 2010/63/EU, Consideration Decree of Scientific Procedures of Animal Experiments (243/1988) and Ethical Codex of Animal Experiments and to the NIH guidelines (Guide for the Care and Use of Laboratory Animals, NIH Publication 86–23). The project was approved by the Ethics Committee on Animal Research of the University of Pécs and license was provided (BA 02/2000–2/2012).

### Serum Transfer Arthritis

K/BxN arthritogenic and BxN non-arthritogenic sera were harvested as described earlier (Jakus et al., 2010). Arthritis was induced by intraperitoneally (i.p.) injecting 150–150 μL of the arthritogenic serum on the 0 and 3 day of the experiment. The control groups received the same amount of non-arthritogenic serum. The mechanonociceptive threshold, heat threshold and cold tolerance were measured (methods described below). Paw edema was quantified using plethysmometry (Ugo Basile 7140), bodyweight was monitored after the first serum administration as a parameter of general well-being. Weight loss was given as the percentage of lost weight compared to pretreatment control values. Arthritis severity was scored using a semiquantitative visual scale where 0–0.5 was no change and 10 was maximal inflammation (Jakus et al., 2010). Swelling and redness of all four feet were considered for the score. To assess joint function mice were placed on a horizontal grid, the grid was turned upside-down and the latency to fall was measured. The grasping ability needed in this test correlates with the joint function. *In vivo* imaging was performed with IVIS Lumina II (PerkinElmer; 60 s acquisition, F/Stop = 1, Binning = 8) before treatment and 2 and 6 days after to quantify plasma extravasation and myeloperoxidase (MPO) activity, as described below. We chose these time points to collect data from the acute phase on day 2 when the vascular inflammatory components are predominant, and from the chronic phase on day 6, when the cellular inflammatory mechanisms are more remarkable (Horváth et al., 2016). On the 14th day after serum administration tibiotarsal joints were removed for histological staining with Safranin stain. Fibroblast proliferation, leukocyte invasion and thickness of synovium were evaluated with 0–3 score depending on severity, the maximum possible score being 9.

### Mast Cell Tryptase (MCT)-Induced Acute Knee Monoarthritis

MCT can be found in abundance in synovial fluid contributing to the inflammation in different types of arthritis (RA, osteoarthritis, spondyloarthritis) by activating the protease-activated receptor 2 (PAR2) on the sensory nerves and inflammatory cells. To investigate acute inflammatory synovial microcirculatory changes, 1 h after guanethidine (12 mg/kg i. p., Sigma) pretreatment, MCT (Merck Millipore) was applied topically to the synovial membrane of the knee joint (12 μg/ml, 20 μL), after removing the skin under ketamine- and xylazine-induced (100 mg/kg and 10 mg/kg i. p, respectively) anesthesia. Contralateral knee joint was treated with 0.9% saline. Blood flow was continuously monitored by laser Speckle imaging for 40 min after the treatment, and the differences compared to the baseline values of the respective area were calculated. To measure acute inflammatory hyperalgesia and edema, MCT was injected intra-articularly (20 μL, 12 μg/ml) into the right knee joint. The paw mechanonociceptive threshold and knee diameter were measured at 2, 4, and 6 h post-injection.

### Complete Freund’s Adjuvant (CFA)-Induced Subacute Knee Monoarthritis

CFA is heat-killed *Mycobacterium tuberculosis* suspended in paraffin oil (1 mg/ml; Sigma-Aldrich) which is taken up by macrophages, activate their reactive oxygen species, cytokine and enzyme generation within a few hours causing localized arthritis of the injected joint without severe systemic symptoms.

CFA (20 μL) was injected into the right mouse knee joint under ketamin-xylazine anesthesia as described above. Contralateral knee joint was treated with 20 μL 0.9% saline. Paw mechanonociceptive threshold and antero-posterior knee diameter were measured 2, 6, and 24 h after CFA administration, changes were expressed as percentage of change compared to the pre-injection values. MPO activity was measured 24 h after treatment.

### Measurement of the Mechanonociceptive and Heat Thresholds

The mechanonociceptive threshold was measured with the dynamic plantar esthesiometer (DPE; Ugo Basile 37000) before (to determine baseline nociceptive threshold) and after treatment. The post-treatment values are shown as the percentage of threshold-decrease of the individual mouse compared to its baseline thresholds. Heat threshold was determined as the temperature where the animal showed nocifensive behavior (shaking, licking, lifting of the hind paw) on increasing temperature hot plate (IITC Life Sciences), the cut-off value was set at 53 °C. Cold hyperalgesia was given as the latency to paw-withdrawal from 0°C water.

### *In vivo* Optical Imaging

Mice were anesthetized with ketamine-xylazine anesthesia for *in vivo* imaging with IVIS Lumina II (PerkinElmer; 120s acquisition, F/stop = 1, Binning = 8) instrument and Living Image^®^ software (PerkinElmer). Luminol sodium salt (5-amino-2,3-dihydro-1,4-phthalazine-dione; 150 mg/kg) injection was given i. p. in sterile PBS solution for MPO imaging. MPO from neutrophil granulocytes produces reactive oxygen species which interact with luminol and result in luminescent signals which we measured 10 min after administration. Luminescence was expressed as total radiance (total photon flux/s) in identical Regions of Interests (ROIs) around the joints.

Inflammatory vascular leakage was evaluated by fluorescence imaging with the Fluorescent Molecular Tomography (FMT) 2000 system (PerkinElmer Ltd.) using the 680 nm laser of the equipment. A micellar formulation of the fluorescent IR-676 dye (Spectrum-Info) dissolved in a 5 w/v% aqueous solution of Kolliphor HS 15 (polyethylene-glycol-15-hydroxystearate; Sigma-Aldrich) was given i. v. in a 0.5 mg/kg dose under ketamine-xylazine anesthesia. The measurement was carried out 20 min afterward dye administration with fluorescence expressed as the calculated amount of fluorophore (pmol) (Botz et al., 2015) in identical Regions of Interests (ROIs) around the ankle joints.

### Tissue Preparation and Protocol for qPCR

L3-L5 lumbar spinal cord and the respective dorsal root ganglia (DRG; total of six DRGs pooled per mouse) were obtained from WT mice 6 days after treatment when behavioral results showed the greatest difference between WT and *Tac4* ^*−/−*^ animals. Mice were divided into three groups: K/BxN treated arthritic (*n* = 8), BxN serum treated control (*n* = 5) and intact control (*n* = 7). Spinal cord samples of *Tac4* ^*−/−*^ mice served as negative (*n* = 3), while inguinal lymph nodes from WT mice served as positive controls (*n* = 2).

Tissue samples were harvested after cervical dislocation and placed immediately into 500 μL TRI reagent (Molecular Research Center, Inc.), snap-frozen on dry ice, then stored on -80°C until processing.

Tissue samples were thawed out on ice and homogenized in TRI reagent. After homegenization, total RNA was extracted using Direct-zol RNA MicroPrep (Zymo Research) according to the manufacturer’s instructions (Aczél et al., 2020). The quantity of the extracted RNA was examined using Nanodrop ND-1000 Spectrophotometer V3.5 (Nano-Drop Technologies, Inc.). RNA samples were treated with dnase I in order to remove contamining genomic DNA, and 500 ng of RNA was reverse transcribed into cDNA using High Capacity cDNA Reverse Transcription Kit (Thermo Fisher Scientific). SensiFAST™ Probe Lo-ROX Kit (Meridiane Bioscience, Memphis, United States) was used according to the manual in the QuantStudio five Real-Time PCR System (Thermo Fisher Scientific). Transcripts of the reference gene *glucuronidase beta* (*Gusb*, Kecskés et al., 2020) and the target gene *Tac4* were evaluated using FAM-conjugated specific probes (Mm01197698_m1, and Mm00474083_m1 respectively, Thermo Fisher Scientific). The gene expression was calculated using ΔΔCt method (Pfaffl, 2001).

### Primary Cultures of Trigeminal Ganglion (TG) Neurons

TG cultures were prepared from neonatal NMRI mice. Ganglia were excised in ice-cold phosphate-buffered saline (PBS), incubated in PBS containing collagenase Type XI (1 mg/ml) and then in PBS with deoxyribonuclease I (1,000 units/ml) for 8 min. Cells were plated on poly-D-lysin-coated glass coverslips in a medium containing Dulbecco’s-Modified Eagle Medium-low glucose (D-MEM), 5% horse serum, 5% newborn calf serum, 5% fetal bovine serum, 0.1% penicillin-streptomycin, 200 ng/ml nerve growth factor (NGF). Cells were maintained at 37°C in a humidified atmosphere with 5% CO2 (Szoke et al., 2010).

### Ratiometric Technique of Intracellular Free Calcium Concentration ([Ca^2+^]i) Measurement with the Fluorescent Indicator Fura-2 AM.

One-two-day-old neurons were incubated for 30 min at 37°C with 1 µM of fluorescent Ca^2+^ indicator dye, fura-2-AM. Cells were washed with extracellular solution (ECS). Calcium transients were detected with microfluorimetry as described elsewhere (Szoke et al., 2010). Fluorescent imaging was performed with an Olympus LUMPLAN FI/x20 0.5 W water immersion objective and a digital camera (CCD, SensiCam PCO) and a Monochromator (Polychrome II., Till Photonics) (generated light: 340 and 380 nm, emitted light: 510 nm). Axon Imaging Workbench 2.1 (AIW, Axon Instruments) software was used, *R* = F340/F380 was monitored, data were subsequently processed by the Origin software version 7.0 (Originlab Corp.). Ratiometric response peak magnitude was measured. Capsaicin (330 nM), AITC (100 µM), HK-1 (500 nM, 1 µM) and SP (500 nM, 1 µM) were administered during the experiments. CP99994, AMG 9810 and HC 030031 were administered in 10 µM concentration.

### Drugs and Chemicals

AITC (Sigma) was dissolved in dimethyl sulfoxide (DMSO) (Sigma) to obtain 10 mM stock solution. Further dilutions were made with ECS solution to reach final concentrations of 100 μM. Capsaicin (Sigma) was dissolved in DMSO to obtain a 10 mM stock solution. Further dilutions were made with ECS or Hank’s solution to reach final concentrations of 330 or 100 nM, respectively. Penicillin-streptomycin was purchased from Gibco. D-MEM-low glucose, collagenase type XI, deoxyribonuclease I, horse serum, newborn calf serum, fetal bovine serum, poly-D-lysine, glycine, NGF, pertussis toxin (PTX), SP, HK-1 were purchased from Sigma. CP99994, AMG 9810 and HC 030031 were purchased from Tocris.

### Statistical Analysis

The treatments were not randomized within cages to prevent control animals from harming the arthritic animals. Results are expressed as the means ± SEM of *n* = 6–10 mice per group in case of *in vivo* functional tests. Data obtained in these experiments were analyzed with repeated measures two-way ANOVA followed by Bonferroni’s post-test with GraphPad Prism 5 software. In all cases *p* < 0.05 was accepted as statistically significant.

## Results

### HK-1 Mediates Mechanical Hyperalgesia, Paw Edema and Decrease in Heat Threshold in Chronic Immune Arthritis

**FIGURE 1:**
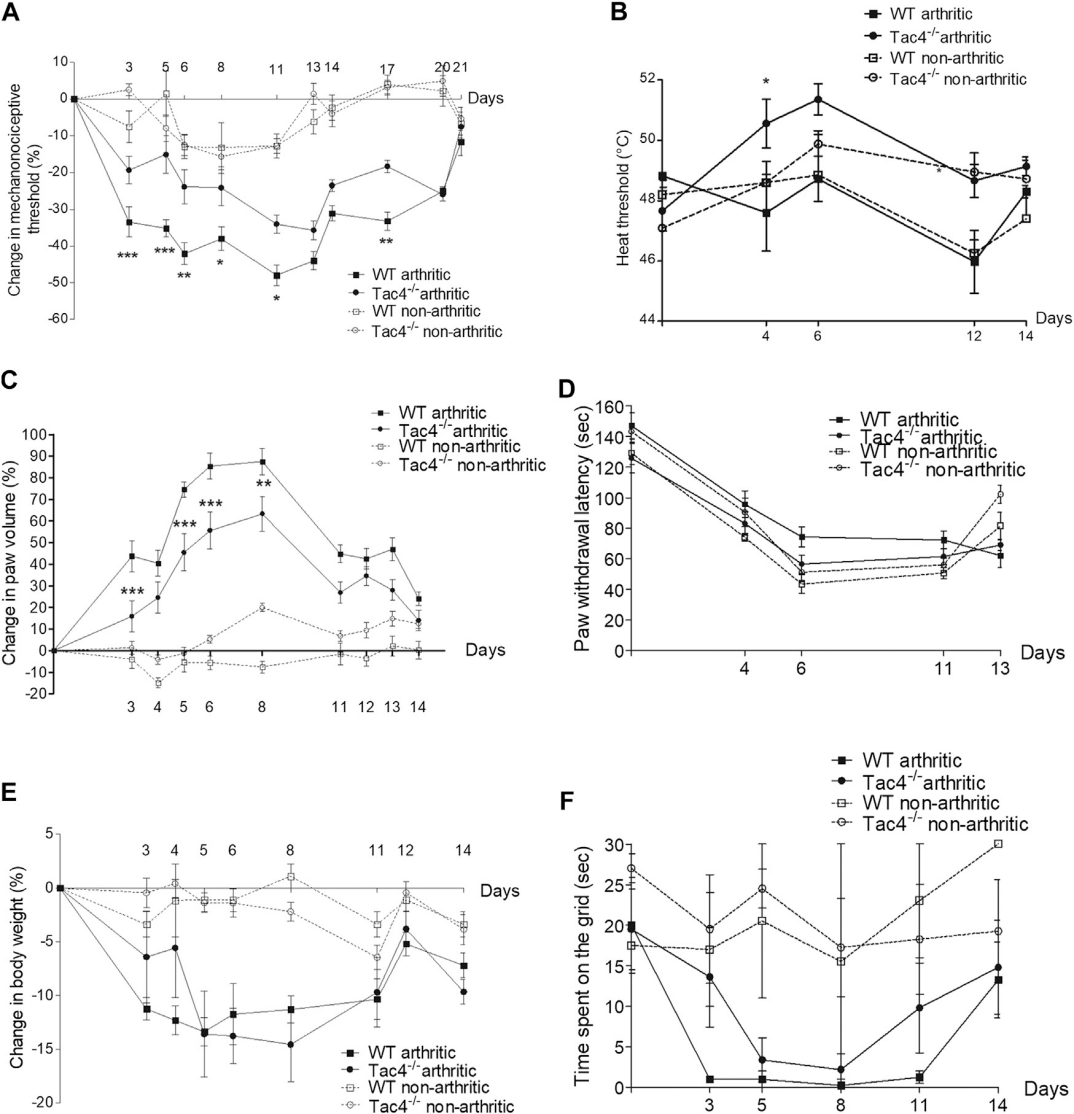
Change in mechanonociceptive threshold **(A)**, heat threshold **(B)**, paw volume **(C)**, cold tolerance **(D)**, body weight **(E)** and time spent on grid **(F)** in K/BxN serum-induced arthritis of wild type (WT) and hemokinin-1-deficient (*Tac4* ^*−/−*^) mice in comparison with BxN serum-treated non-arthritic groups (*n* = 4–10 per group; *p < 0.05, **p < 0.01, ***p < 0.001 vs. arthritic WT, repeated measures two-way ANOVA + Bonferroni’s post test).

Compared to pre-treatment control values, a significant decrease in pain threshold developed in WT mice by the 5th day of the experiment (-33.5 ± 4.1%). This spontaneously resolved by the end of the 3rd week. *Tac4* ^*−/−*^ mice showed significantly milder decrease in the pain threshold (-19.4 ± 3.8% on day 5) throughout the entire experiment (Figure 1A). The decrease in heat threshold began in WT mice at the 4th day (47.6 ± 1.3°C) and was significantly less severe in *Tac4* ^*−/−*^ mice (50.6 ± 0.8°C) (Figure 1B).

Paw edema developed by the 3rd day of the experiment (43.8 ± 7.2% in WT), and spontaneously resolved by 2 weeks after serum administration with significantly milder edema seen in *Tac4* ^*−/−*^ mice (Figure 1C). Decrease in cold tolerance, body weight and time spent on grid occurred in the experiment, but gene deletion resulted in no difference (Figures 1D–F). Change in mechanonociceptive threshold, arthritis score, paw volume, cold tolerance, body weight and time spent on grid in NK1R deficient mice showed no difference compared to WT mice (Supplementary Figure S1).

### HK-1 Decreases MPO-Activity in K/BxN Serum-Transfer Arthritis

**FIGURE 2:**
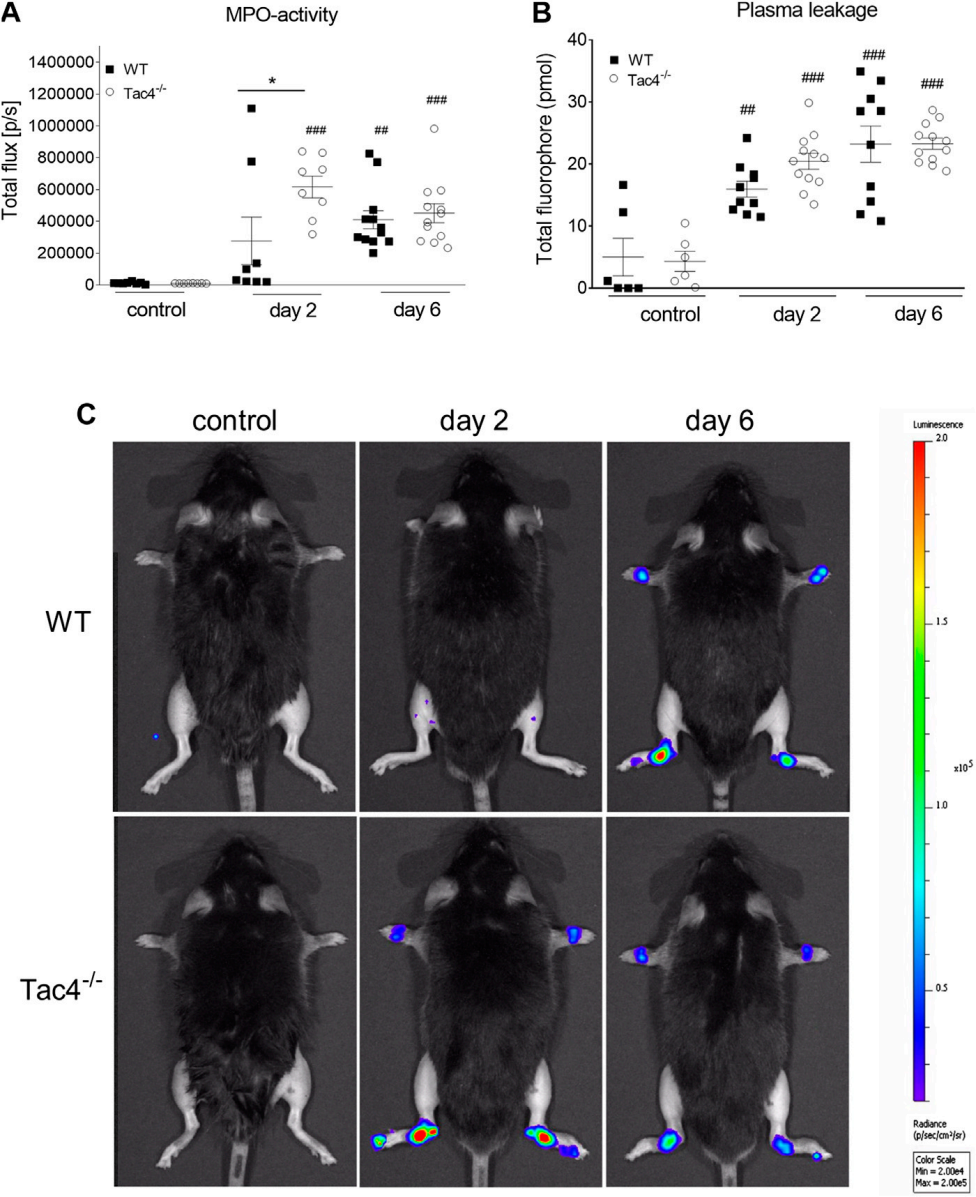
Quantitative evaluation of MPO-activity **(A)** and IR-676 dye extravasation **(B)** in K/BxN arthritogenic serum-treated wild type (WT) and hemokinin-1-deficient (*Tac4* ^−/−^) mice. Representative images of MPO-activity **(C)**. Box plots demonstrate medians with the upper and lower quartiles and all individual data points; *n* = 6–12 per group; *p < 0.05 vs. WT, ##*p* < 0.01, ###*p* < 0.001 vs. control (one-way ANOVA + Bonferroni’s post test).

*Tac4* ^*−/−*^ mice showed a marked increase in MPO-activity 2 days after serum administration (6,16 × 105 ± 6,83 × 104 *p*/s), whereas in WT mice it became significant on the 4th day (409,917 ± 56729) (Figure 2A). An increase in plasma extravasation was detectable in both groups on the 2nd and 6th day, but no effect of the gene-deletion could be observed (Figure 2B). Representative pictures of MPO-activity can be seen on Figure 2C.

### HK-1 Increases Histopathological Arthritis Severity

**FIGURE 3:**
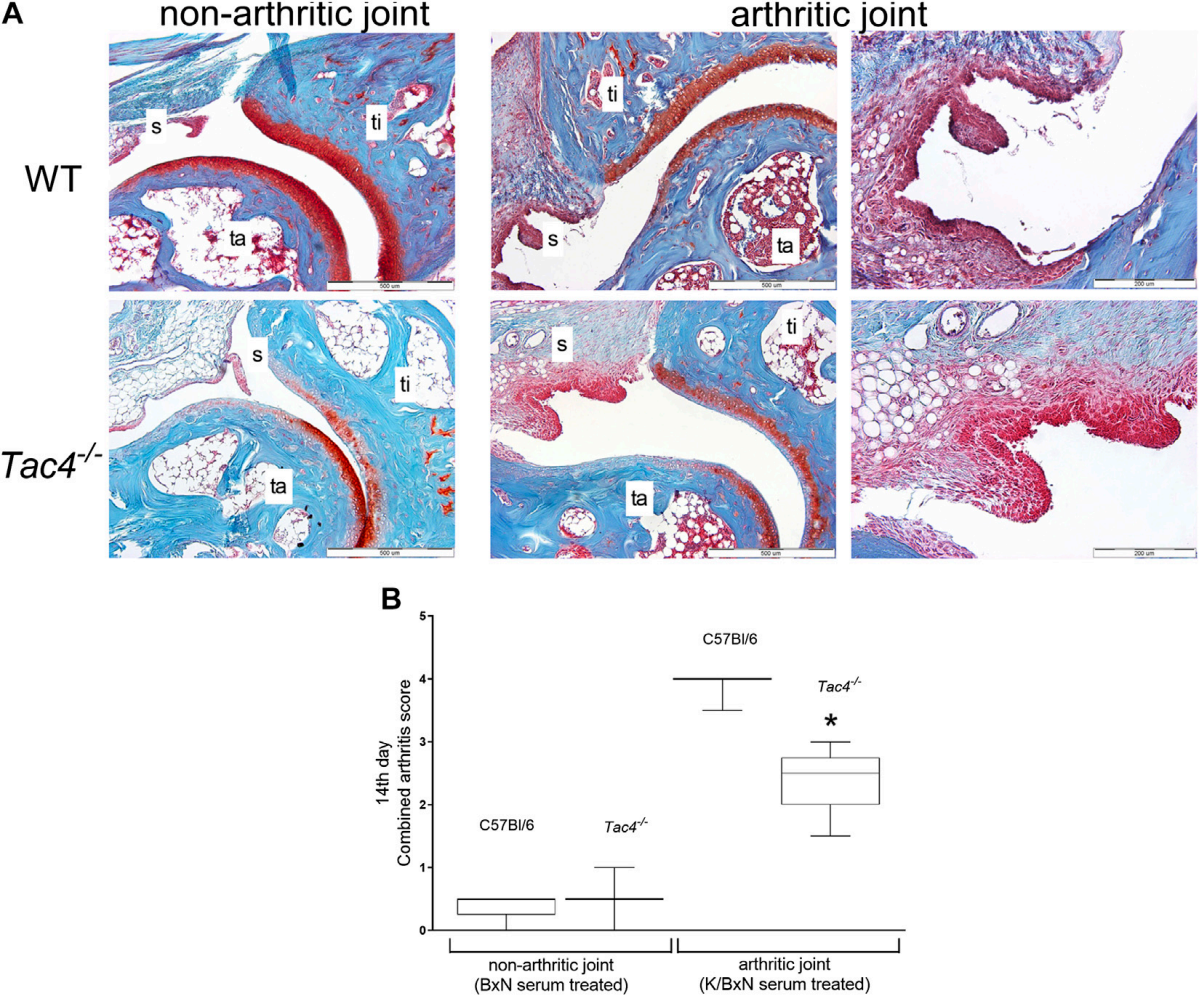
Representative histopathological pictures of safranin O-stained tibiotarsal joint showing ×100 and ×200 magnifications with 500 and 200 µm scale bars, respectively. ti = tibia, ta = tarsus, s = synovium **(A)**. Semiquantitative histopathological score of K/BxN serum treated wild type (WT) and hemokinin-deficient (*Tac4* ^−/−^) arthritic mice in comparison with non-arthritic controls. Box plots demonstrate the medians with upper and lower quartiles of n = three to five per group (*p < 0.05 vs. WT, Mann-Whitney test) **(B)**.

On the 14th day of the experiment WT mice had a severity score of 4.0 ± 0.5 out of a maximum of nine points, while *Tac4* ^*−/−*^ mice had a significantly lower score of 2.5 ± 0.5 (Figure 3).

### *Tac4* mRNA Expression in DRG and Spinal Cord

*Tac4* mRNA showed a stable expression in the DRG throughout the experiment, with a slight, non-significant decrease on day 6 (Supplementary Figure S3). In dorsal spinal cord samples, *Tac4* specific signals were not detectable. The specificity of the reaction was proved by using inguinal lymph nodes as positive control. RNA samples of *Tac4* ^*−/−*^ mice served as negative controls, in which samples we did not observe any amplification products with the applied probes.

### HK-1 Mediates Mechanical Hyperalgesia and Knee Edema in MCT-Induced Acute Monoarthritis

**FIGURE 4:**
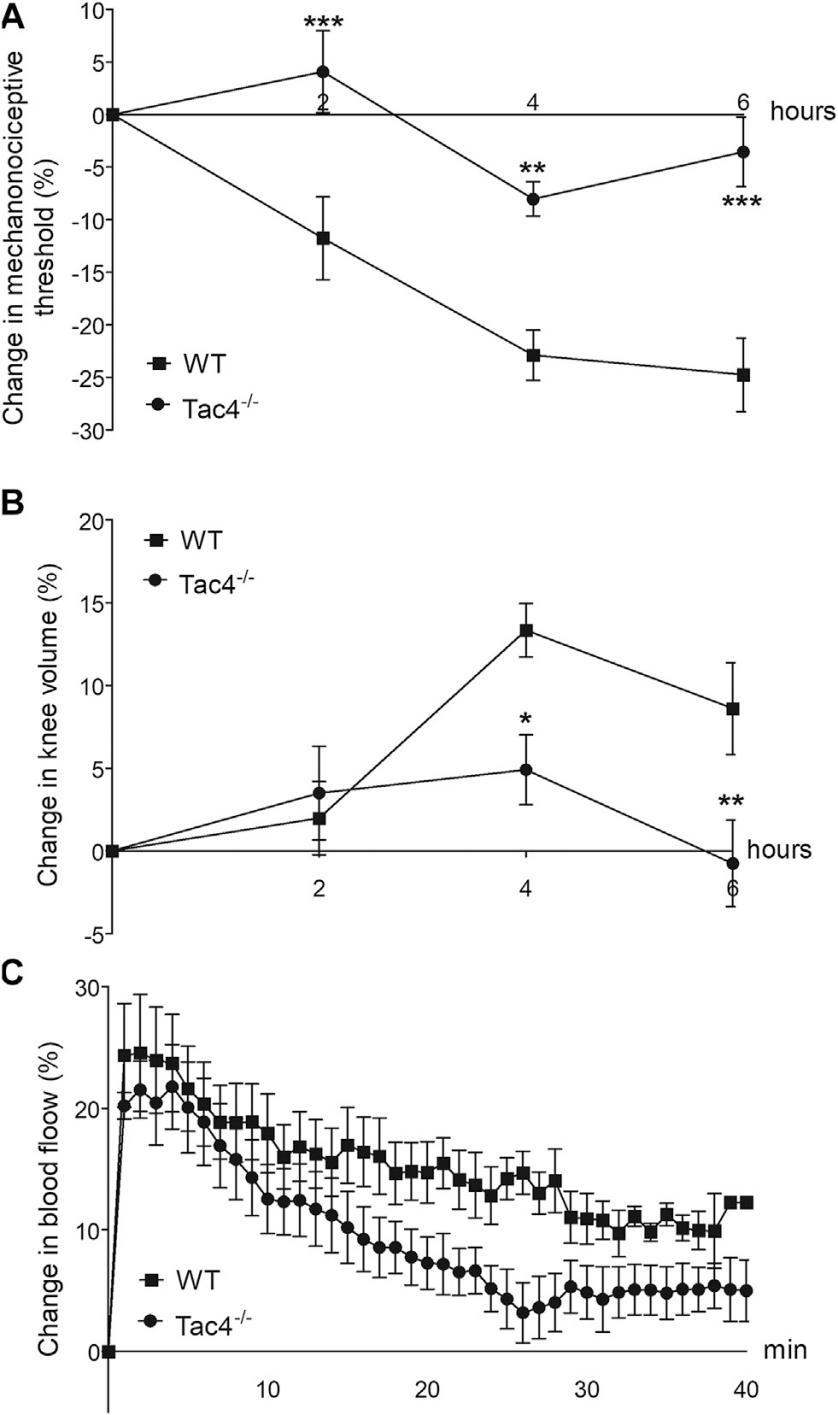
Changes in mechanonociceptive threshold **(A)**, knee diameter **(B)** and blood flow **(C)** in mast cell tryptase-induced acute arthritis of wild type (WT) and hemokinin-1-deficient (*Tac4* ^−/−^) mice. Data points demonstrate the means ± SEM of *n* = 5–10 per group; *p < 0.05, **p < 0.01, ***p < 0.001 vs. WT (repeated measures two-way ANOVA + Bonferroni’s post test).

Mechanical hyperalgesia developed in WT mice 2 days after MCT administration (-11.8 ± 4.0%), while knee edema developed on the 4th day (13.3 ± 1.6). Both parameters were significantly less severe in *Tac4* ^*−/−*^ mice. Increase in blood flow was detectable in the first 40 min after treatment but showed no significant difference between the groups (Figure 4).

### HK-1 Mediates Mechanical Hyperalgesia and Knee Edema, but Decreases MPO Activity in CFA-Induced Subacute Knee Inflammation

**FIGURE 5:**
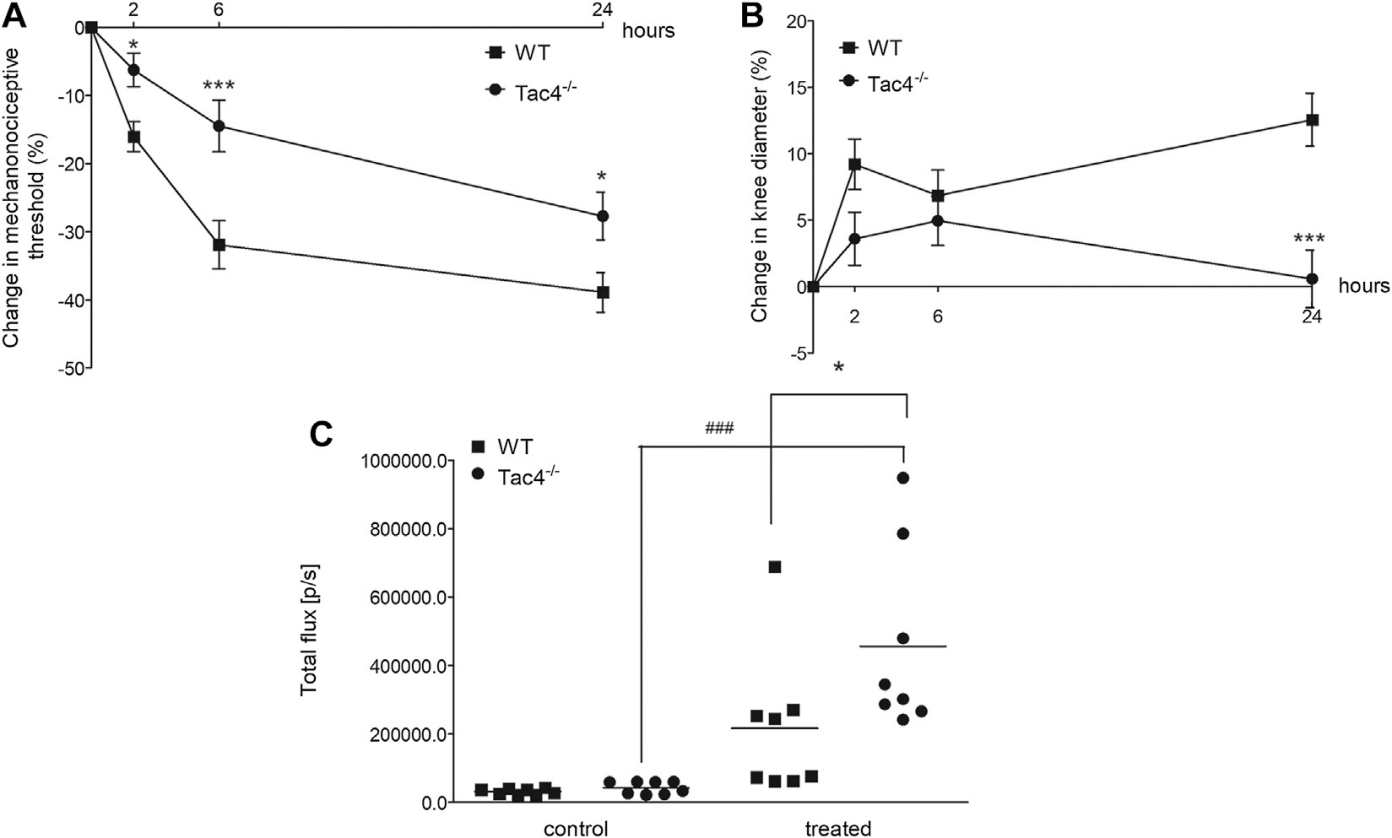
Changes in mechanonociceptive threshold **(A)**, AP knee diameter **(B)**, and MPO-activity **(C)** in CFA-induced acute arthritis of WT and HK-1-deficient (*Tac4* ^*−/−*^) mice. Data points demonstrate the means ± SEM **(A)**, **(B)** or medians with upper and lower quartiles of *n* = 8–15 per group; *p < 0.05, **p < 0.01, ***p < 0.001 vs. WT, ###*p* < 0.001 vs. control (repeated measures two-way ANOVA + Bonferroni’s post test; for MPO activity: one-way ANOVA + Bonferroni’s post test).

Mechanical hyperalgesia and knee edema were detectable 2, 6 and 24 h after the CFA administration. *Tac4* ^*−/−*^ mice had a significantly milder mechanical hyperalgesia at every time point and less severe knee edema at 24 h. *Tac4* ^*−/−*^ mice showed a significant increase in MPO-activity (457,125 ± 94397) (Figure 5). Changes in mechanonociceptive threshold and knee volume did not show significant difference to WT mice in NK1R deficient mice (Supplementary Figure S2).

### HK-1 Directly Activates Primary Nociceptive Sensory Neurons

**FIGURE 6:**
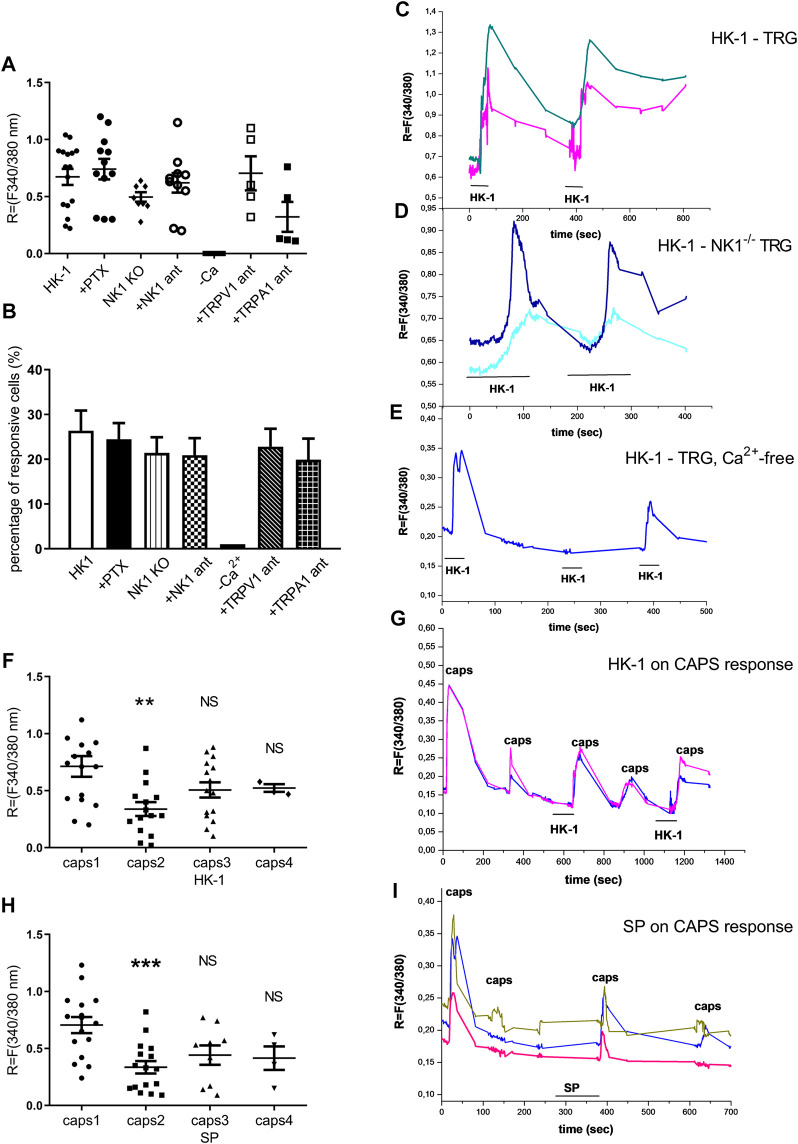
Effect of HK-1 and SP in cultured primary sensory neurons. Change in the fluorescence ratio (*R* = F340/F380) in HK-1-sensitive cells (•) is presented. ●: HK-1 and PTX co-administration, ♦: fluorescence signal in neurons from *NK1R* ^*−/−*^ animals, ○: HK-1 and CP99994 NK1 receptor antagonist administration, □: HK-1 and AMG9810 TRPV1 receptor antagonist, ■: HK-1 and HC 030031 TRPA1 receptor antagonist. No signal was detected in Ca^2+^-free extracellular solution. *N* = 19–49 cells/group **(A)**. The percentage of responsive cells to HK-1, HK-1+PTX, HK-1 in Ca^2+^-free condition, HK-1+NK1 receptor antagonist CP99994 and HK-1 in TG from *NK1R* ^*−/−*^ animals, HK-1 + TRPV1 receptor antagonist AMG9810 and HK-1 + TRPA1 receptor antagonist HC 030031 is presented. Ca^2+^-responses are presented in % of total number of examined neurons. *N* = 19–49 cells per group **(B)**. Original Ca^2+^-imaging registrations after HK-1 administration. Increases of *R* = 340/380 fluorescence in fura-2 loaded cultured TG neurons **(C)**. Original Ca^2+^-imaging registrations after HK-1 administration in TG neurons from NK1 gene deficient mice **(D)**. Original Ca^2+^-imaging registrations after HK-1 administration in Ca^2+^-free solution **(E)**. Effect of HK-1 on capsaicin-induced Ca^2+^-influx. Increases of *R* = 340/380 fluorescence in fura-2 loaded neurons are presented, **p < 0.01, NS (vs. caps1, one-way ANOVA with Bonferroni’s multiple comparison post hoc test, *n* = 16) **(F)**. Effect of HK-1 on capsaicin-induced Ca^2+^-influx. Original Ca^2+^-imaging registrations after capsaicin and HK-1 administration **(G)**. Effect of SP on capsaicin-induced Ca^2+^-influx. Increases of *R* = 340/380 fluorescence in fura-2 loaded neurons are presented, ***p < 0.001, NS (vs. caps1, one-way ANOVA with Bonferroni’s multiple comparison post hoc test, *n* = 17) **(H)**. Effect of SP on capsaicin-induced Ca^2+^-influx. Original Ca^2+^-imaging registrations after capsaicin and SP administration **(I)**.

First, the effects of HK-1 and SP were investigated. Both peptides in 500 nM (Figures 6A–E) and SP in 1 µM concentration (data not shown) had no effect on Ca^2+^-influx, but 1 μM HK-1 caused remarkable Ca^2+^-influx (R = 0.67 ± 0.07) in 26.39 ± 4.5% of the neurons (19 out of 72). In the next step, we investigated the mechanism of HK-1 action and the characteristics of the Ca^2+^-signal. The NK1 receptor antagonist CP99994 did not influence the HK-1 response, 20.93 ± 3.8% of the cells (9 out of 43) responded with Ca^2+^-influx (R = 0.62 ± 0.08). This was similar in neurons of NK1R gene-deleted mice (21.4 ± 3.5%, nine out of 42 responsive cells, R = 0.49 ± 0.04). The G-protein-coupled receptor (GPCR) blocker PTX influenced neither the ratio of the responding neurons (24.49 ± 3.6%; 12 out of 49) nor the extent of the response (R = 0.74 ± 0.03) to HK-1. In order to investigate potential HK-1-induced Ca^2+^-release from intracellular stores as a consequence of PTX-insensitive GPCR mechanism, we did measurements using Ca^2+^ free ECS. No Ca^2+^-signal was detected in this condition indicating that HK-1 evokes Ca^2+^-influx from the extracellular space. The response to HK-1 was detected in the presence of the TRPV1 antagonist AMG8910, 22.7 ± 4% of the cells (5 out of 22) responded with Ca^2+^-influx (R = 0.7 ± 0.28) and the TRPA1 antagonist HC 030031, 19.2 ± 4.7% of the cells (5 out of 26) responded with Ca^2+^-influx (R = 0.32 ± 0.26).

In the next experiment four or five repeated treatments of capsaicin on the same cultured sensory neurons were performed, and the effect of 500 nM HK-1 and SP was investigated on capsaicin-evoked Ca^2+^-influx. The first application of 330 nM capsaicin induced transient Ca^2+^-accumulation which gradually decreased in response to the second capsaicin stimulus due to TRPV1 (transient receptor potential cation channel subfamily V member 1) desensitization. Meanwhile, both HK-1 and SP administered in separate cultures after the second capsaicin stimulus diminished the desensitization as shown by the third and fourth capsaicin-evoked responses (Figures 6F–I).

## Discussion

Here we provide the first evidence for an important role of HK-1 in pain transmission using different arthritis models. This is likely to be mediated by direct activation of primary sensory neurons via NK1R-independent, PTX-insensitive, but extracellular Ca^2+^-dependent mechanism. Besides its key importance in pain development, HK-1 has a complex regulatory function in joint inflammatory processes: it mediates edema formation and histopathological alterations including inflammatory cell accumulation, but inhibits early neutrophil/macrophage-dependent MPO-activity increase in the chronic model.

K/BxN induced arthritis is a widely accepted chronic passive transfer disease model (Malcangio, 2020), MCT is an important local mediator of inflammation (Kobayashi and Okunishi, 2002), mediating its effects through sensory neurons (Borbély et al., 2016), and acute CFA (Billiau and Matthys, 2001) induced arthritis is initiated by macrophages. Using these three methods we could obtain a more detailed picture on how HK-1 influences the development of joint inflammation and related pain.

The deficiency of HK-1, but not the NK1R resulted in significantly decreased swelling in the acute models and also in the early phase of the chronic experiment. Plasma leakage was not altered by HK-1 on days 2 and 6, but only venular fenestration and dye extravasation at the timepoints of the examination can be detected with the applied *in vivo* fluorescent imaging technique (Botz et al., 2015). This is one component of the edema formation, but it does not exclusively explain paw swelling differences at the respective observation timepoints mainly due to different kinetics of the different components of the vascular changes (vasodilation and leakage). We found no difference in edema formation in the chronic adjuvant-induced active immunization-based model in HK-1 or NK1R deficient mice (Borbély et al., 2013), therefore, the edemogenic action of HK-1 seems to develop in a much earlier phase of the inflammatory process. The arteriolar vasodilation and microcirculation-increase was not affected either by HK-1-deletion in the MCT-model, which suggests that HK-1 is not a predominant regulator of the vascular (Borbély et al., 2013) functions.

Cellular inflammatory response in the chronic arthritis model, as shown by the histopathological arthritis score, was significantly reduced in HK-1-deficient mice. These results are supported by the well-established immunoregulatory role of HK-1. B-cells play an important role in RA, restricting their function has been shown to ameliorate autoimmune arthritis (Tóth et al., 2019), and HK-1 plays a critical role in the development of these cells (Zhang et al., 2000). It also influences monocyte/macrophage development (Berger et al., 2007) and neutrophils *in vitro* (Klassert et al., 2008), which play a role in arthritis development (Smith and Haynes, 2002). We found that despite reduced functional and morphological inflammatory alterations, MPO increase related to neutrophil and macrophage activation occurred earlier (on day 2) in the absence of HK-1. This virtual contradiction can be explained by data showing that although MPO is generally known as a mediator of tissue damage and inflammation, it has been shown to prevent inflammation as well (Arnhold and Flemmig, 2010). The elevation of MPO especially in the early phase of the inflammatory cascade is considered to be protective, though the mechanism is not well understood. MPO products have been suggested to downregulate innate immunity, facilitate the switch to adaptive immunity and inhibit T-cell reponses (Prokopowicz et al., 2012). We have seen similar relation between elevated MPO and decreased inflammatory parameters in a previous study (Horváth et al., 2016). Since HK-1 and MPO are both produced by neutrophils, they might have direct interactions, but these have not yet been studied. There is limited evidence about the involvement of HK-1 in pain which, in agreement with our present results, found it to be pro-nociceptive. Intracerebroventricular administration of low dose (1–10 pmol) HK-1 caused nocifensive behavior, while high dose (≥0.1 nmol) caused analgesia, all of which could be counteracted by an NK1R antagonist, as well as opioid antagonists (Fu et al., 2005). Other studies have shown that lumbar intrathecal administration of 0.1 nmol HK-1 caused pain reaction in mice which could be inhibited by an NMDA receptor antagonist (Watanabe et al., 2016), but not an NK1R antagonist (Watanabe et al., 2010). These results suggest that high doses of HK-1 might have non-specific actions resulting in divergent outcomes. Our earlier paper provided evidence that HK-1 has an important role in neuropathic pain and microglia activation (Hunyady et al., 2019). Other studies found that HK-1 mRNA expression increases in the dorsal spinal cord of neuropathic rats which could be blocked by inhibiting microglia activation and alleviating pain (Matsumura et al., 2008). Our current results show that HK-1 mediates both the early inflammatory pain, and the late neuropathic-type pain in arthritis observed during the 3rd week of the K/BxN experiment (Christianson et al., 2010), while other studies showed the activation of spinal microglia in experimental arthritis (Agalave et al., 2014). Earlier findings showed significantly decreased HK-1 mRNA in the DRG in the collagen antibody induced arthritis (CAIA) mouse model (Makino et al., 2012), which is in agreement with the tendency we demonstrated in the present paper, although the decrease was not statistically significant due to individual variations. Joint inflammation was alleviated by NK1R antagonists in the CAIA model, while the pain could only be inhibited by indomethacin. In agreement with these findings we have also showed that the NK1 receptor does not play a role in arthritic pain, however, we conclude that HK-1 might be an important mediator in an NK1R-independent manner by directly activating primary sensory neurons.

Despite little information about HK-1 in pain and arthritis, the best-known member of the tachykinin family, SP, and the NK1R have been thoroughly investigated in these conditions (Zieglgänsberger, 2019). It is well established that SP via NK1R activation is involved in pain, which initiated considerable pharmacological research in this field. Despite the proof-of-concept of SP as a pain mediator, unfortunately, NK1R-antagonists have failed as analgesics in clinical trials (Botz et al., 2017), suggesting a yet unknown mechanism that can circumvent NK1R-targeted approaches. SP mediates chondrocyte differentiation in cell cultures (Millward-Sadler et al., 2003) and vasodilation in arthritic mice (Keeble et al., 2005) through the NK1 receptor. SP-like immunoreactivity was shown to increase in the primary sensory neurons of dorsal root ganglia (DRG) of arthritic mice (Willcockson et al., 2010), but the number of sensory nerve fibers of the arthritic joint capsule, that are the main sources of SP, decreased (Buma et al., 2000). Since SP and HK-1 cannot be differentiated by immunological methods, it is possible that these immunohistochemistry data referred (at least partially) to HK-1 expression.

In order to explore the direct effect and mechanism of action of HK-1, we did further studies on cultured primary sensory neurons. HK-1, but not SP, induced Ca^2+^-influx into these neurons. HK-1-induced direct Ca^2+^-influx could be seen during PTX administration and also in sensory neurons derived from *NK1R* ^*−/−*^ animals. The main finding of our experiments was that the Ca^2+^-influx was coming from the extracellular space in an NK1R-independent manner since the signal disappeared in Ca^2+^-free extracellular solution. It suggests an ion channel-coupled receptorial mechanism, but not TRPV1- or TRPA1-mediated mechanism, since the TRPV1 receptor antagonist AMG9810 and the TRPA1 receptor antagonist HC 030031 did not influence the HK-1-induced Ca^2+^ influx response. Furthermore, even lower concentrations of both HK-1 (similarly to SP) diminished desensitization of the TRPV1 receptor that upon repeated capsaicin administration could also contribute to the peripheral pain generating and maintaining effect of HK-1. The neuronal activating potential of HK-1 is supported by earlier data showing the ability of HK-1 to evoke direct post-synaptic activation of cholinergic hippocampal neurons in a tetrodotoxin-resistant manner. However, unlike sensory neurons, this response was not inhibited in Ca^2+^-fee media and was similar to the effect of SP (Morozova et al., 2008).

Some limitations of the present study are that we only performed our experiments on male mice to avoid the influencing factors of the estrus cycle and that we could detect HK-1 neither qualitatively by immunohistochemistry nor quantitatively by immune assays due to the lack of antibodies able to differentiate it from SP.

In conclusion, we provide the first evidence using three different mouse models mimicking distinct mechanisms of rheumatoid arthritis and integrative methodology (functional, *in vivo* optical imaging, cell cultures) that HK-1 mediates several arthritic inflammatory mechanisms and pain by direct activation of primary sensory neurons not via its classical NK1 receptor. The main translational value is that we modeled the late neuropathic component of arthritic pain related to neuropathic mechanisms without inflammatory symptoms and describe the involvement of HK-1 in this process. These findings add to the growing evidence that HK-1 has another, so far unidentified target initiating further research in this direction.

## Data Availability Statement

The original contributions presented in the study are included in the article/Supplementary Material, further inquiries can be directed to the corresponding author.

## Ethics Statement

The animal study was reviewed and approved by Ethics Committee on Animal Research of the University of Pécs.

## Author Contributions

All authors were involved in drafting the article or revising it critically for important intellectual content, and all authors approved the final version to be published. ZH and AH had full access to all the data in the study and take responsibility for the integrity of the data and the accuracy of the data analysis. ÉB and AH, and also ÉS and ZH contributed equally to this work, respectively.

## Funding

This work was supported by EFOP‐3.6.2‐16‐2017‐00008 “The role of the neuroinflammation in neurodegeneration: from molecules to clinics”, 2017‐1.2.1‐NKP‐2017‐00002 (NAP‐2; Chronic Pain Research Group), EFOP‐3.6.1.‐16‐2016‐0004 and GINOP 2.3.2‐15‐2016‐00050 “PEPSYS”. ÉB 2019 and ÉS 2017 were supported by the János Bolyai Research Scholarship of the Hungarian Academy of Sciences. KP was supported by GYTK‐KA‐2020‐01, University of Pécs Faculty of Pharmacy and the New National Excellence Program of the Ministry for Innovation and Technologies from the source of the National Research, Development and Innovation Fund ÚNKP‐20‐4‐II‐PTE‐465. BB 2019 was supported by the János Bolyai Research Scholarship of The Hungarian Academy of Sciences and the ÚNKP‐20‐5‐PTE‐540 New National Excellence Program of the Ministry for Innovation and Technology. The University of Pécs is acknowledged for a support from the 17886‐4/23018/FEKUTSTRAT excellence grant. AM was supported by the “Élvonal” program from the Hungarian National Agency for Research, Development and Innovation (KKP 129954).

## Conflict of Interest

Zsuzsanna Helyes is the strategic director and shareholder of PharmInVivo Ltd. (Pécs, Hungary) and shareholder of Algonist Biotechnologies Gmbh, (Wien, Austria). Eva Szoke is also a shareholder of Algonist Biotechnologies Gmbh, (Wien, Austria), but there is no conflict of interest with the present work. These companies were not involved in the study design, funding, collection, analysis, interpretation of data, the writing of this article or the decision to submit it for publication.

The remaining authors declare that the research was conducted in the absence of any commercial or financial relationships that could be construed as a potential conflict of interest.

## Glossary

AITC: allyl isothiocyanate
CAIA: collagen antibody induced arthritis
CFA: complete Freund’s adjuvant
DMARD: disease-modifying antirheumatic drugs
D-MEM: Dulbecco’s-Modified Eagle Medium-low glucose
DMSO: dimethyl sulfoxide
DPE: dynamic plantar esthesiometer
DRG: dorsal root ganglion
ECS: extracellular solution
GPCR: G-protein-coupled receptor
HK-1: hemokinin-1
i.p.: intraperitoneal
i.v.: intravenous
KO: knockout
MCT: mast cell tryptase
MPO: Myeloperoxidase
NGF: nerve growth factor
NK1R: tachykinin neurokinin-1 receptor
NK2R: tachykinin neurokinin-2 receptor
NK3R: tachykinin neurokinin-3 receptor
NMDA: N-metil-d-aspartic acid
NSAID: nonsteroidal anti-inflammatory drugs
PAR2: protease-activated receptor two
PBS: phosphate-buffered saline
PCR: Polymerase chain reaction
PTX: pertussis toxin
RA: rheumatoid arthritis
ROI: region of interest
SP: substance P
Tac4: preprotachykinin-4 gene
Tacr1: tachykinin receptor one gene, encodes NK1R
TG: trigeminal ganglion
TRPA1: transient receptor potential cation channel subfamily A member one
TRPV1: transient receptor potential cation channel subfamily V member one
WT: wild type
