# Supplementary figures and images for: Hemokinin-1 as a Mediator of Arthritis-Related Pain via Direct Activation of Primary Sensory Neurons
Source: Front Pharmacol. 2021 Jan 13;11:594479. doi: 10.3389/fphar.2020.594479 (PMC7839295; doi:10.3389/fphar.2020.594479)

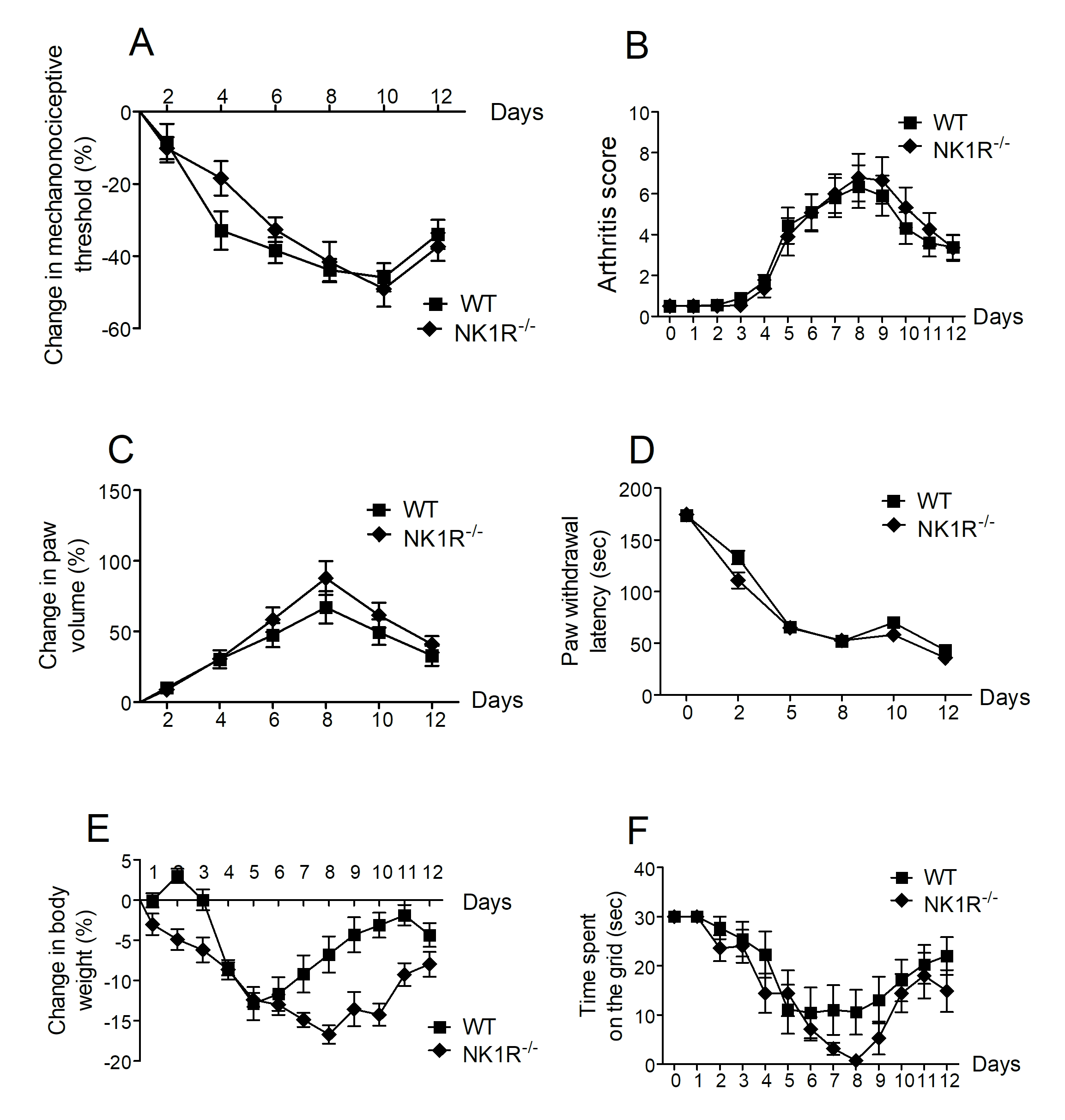

Supplement: Supplementary file 1 [file image1.tif]

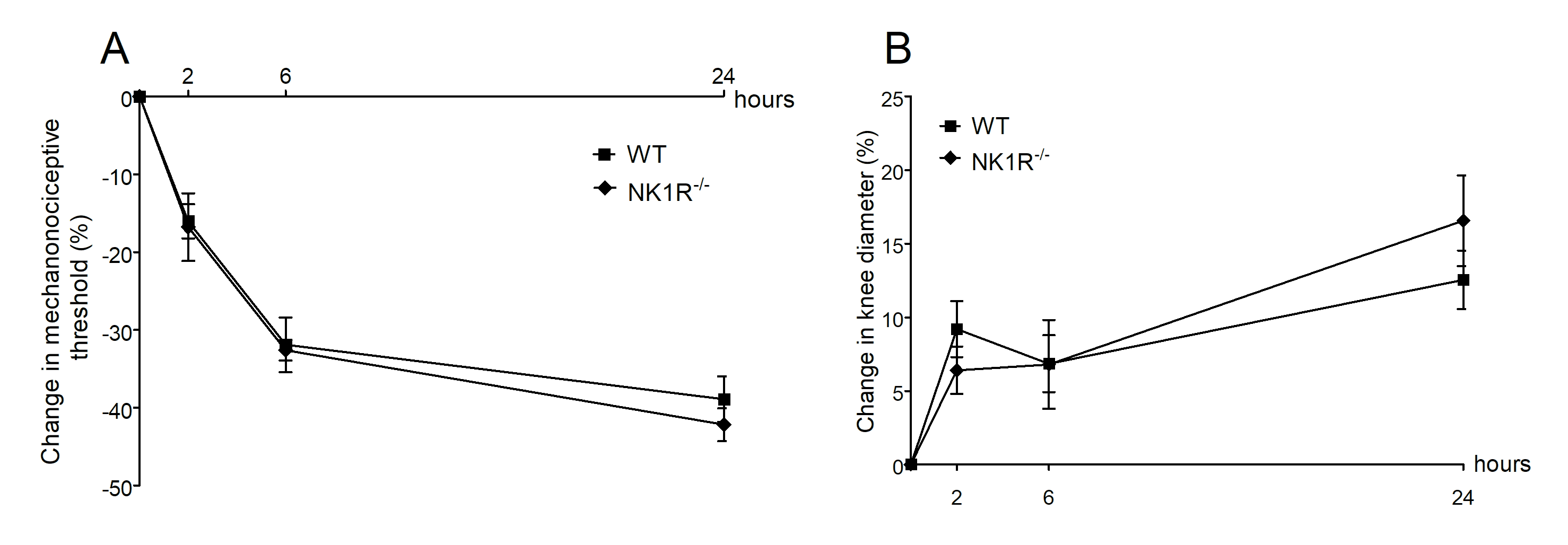

Supplement: Supplementary file 2 [file image2.tif]

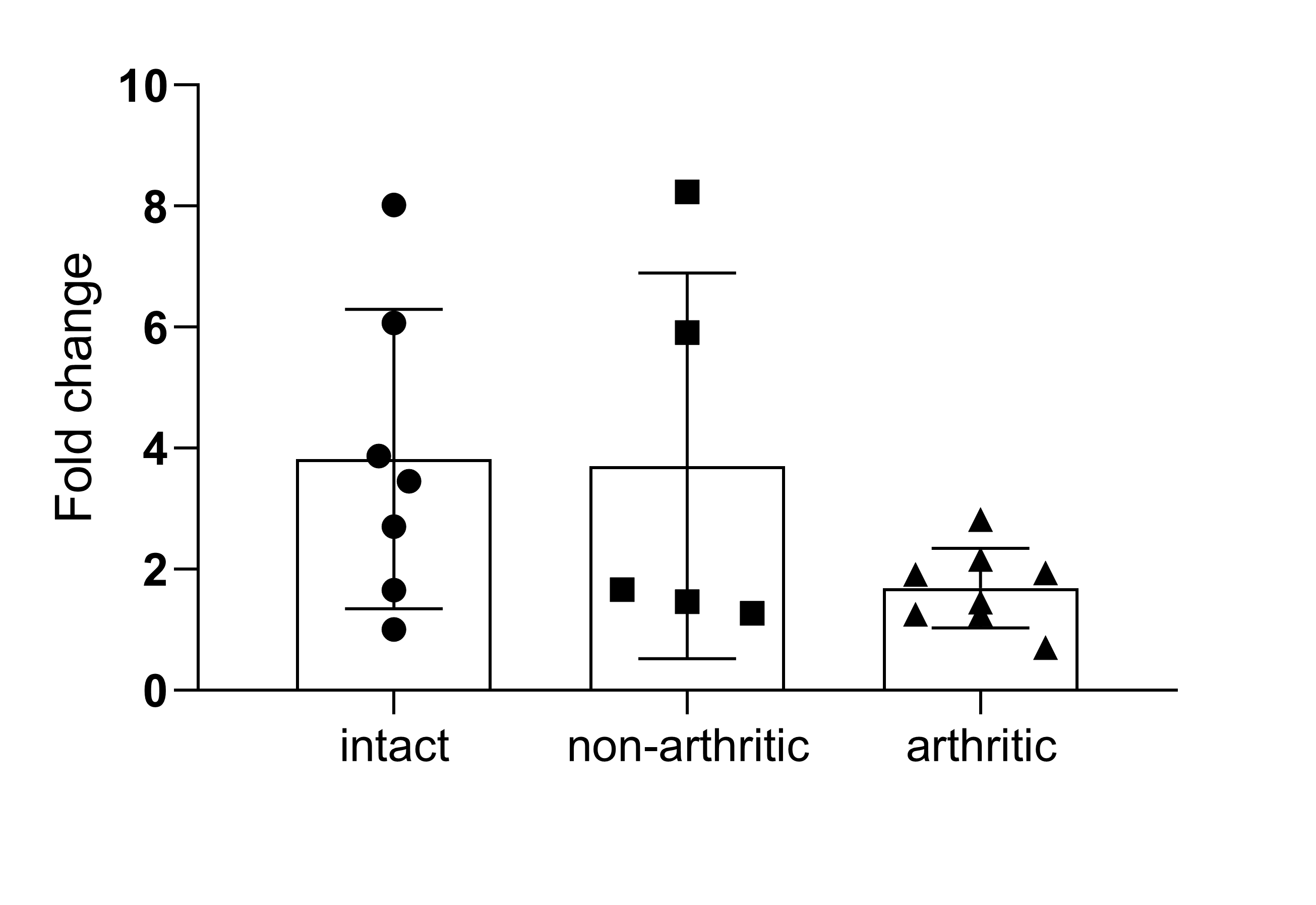

Supplement: Supplementary file 3 [file image3.tif]
